# Correlation and anti-correlation of the East Asian summer and winter monsoons during the last 21,000 years

**DOI:** 10.1038/ncomms11999

**Published:** 2016-06-22

**Authors:** Xinyu Wen, Zhengyu Liu, Shaowu Wang, Jun Cheng, Jiang Zhu

**Affiliations:** 1 Department of Atmospheric and Oceanic Sciences & Laboratory for Climate and Ocean-Atmosphere Studies, School of Physics, Peking University, Beijing 100871, China; 2 Department of Atmospheric and Oceanic Sciences & Center for Climatic Research, Nelson Institute for Environmental Studies, University of Wisconsin-Madison, WI 53706, USA; 3 Polar Climate System and Global Change Laboratory, Nanjing University of Information Science and Technology, Nanjing 210044, China

## Abstract

Understanding the past significant changes of the East Asia Summer Monsoon (EASM) and Winter Monsoon (EAWM) is critical for improving the projections of future climate over East Asia. One key issue that has remained outstanding from the paleo-climatic records is whether the evolution of the EASM and EAWM are correlated. Here, using a set of long-term transient simulations of the climate evolution of the last 21,000 years, we show that the EASM and EAWM are positively correlated on the orbital timescale in response to the precessional forcing, but are anti-correlated on millennial timescales in response to North Atlantic melt water forcing. The relation between EASM and EAWM can differ dramatically for different timescales because of the different response mechanisms, highlighting the complex dynamics of the East Asian monsoon system and the challenges for future projection.

## Abstract (web summary)

Future projection of changes in the East Asia Summer and Winter Monsoon are hindered by a lack of understanding of past variability. Here, using longterm transient simulations, the authors show that the monsoons respond in phase to precessional forcing, yet out of phase millennial-scale North Atlantic forcing.

## Graphical abstract

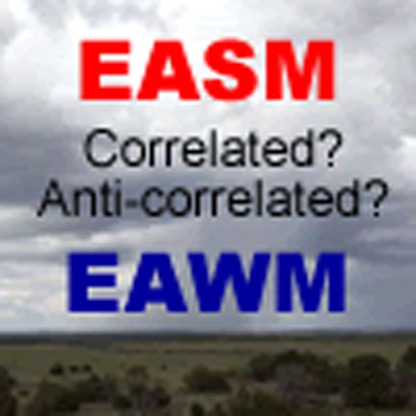

The East Asia monsoon system consists of a southwesterly summer monsoon and a northerly winter monsoon. The co-variability of EASM and EAWM on multiple timescales significantly affect East Asia's climate in the past and future^1^,^2^. The last decade has seen an explosive growth of high-resolution proxy records of EASM^3^,^4^,^5^,^6^,^7^,^8^,^9^,^10^,^11^,^12^ during the glacial-interglacial cycles, notably from cave δ^18^O records in southeastern China and nearby regions^3^,^4^,^5^,^6^,^7^,^8^,^9^,^13^. These records consistently suggest an EASM evolution that follows the Northern Hemisphere summer insolation while punctuated by abrupt millennial events. In comparison, high-resolution proxy records are less abundant for EAWM^14^,^15^,^16^,^17^ and the records available seem to show inconsistent results^14^,^16^,^17^,^18^,^19^, leaving a debate on the co-variability between the EASM and EAWM ambiguous. The first high-resolution EAWM index constructed with Titanium content in the sediment of Lake Huguang Maar in South China suggested an anti-correlation between the EASM and EAWM over the past 16,000 years, especially over the major millennial periods of Bølling-Allerød (BA), Younger Drays (YD) and the mid-to-late Holocene^14^. However, the lake sediment record has been argued to be a proxy for local hydrology rather than the large-scale EAWM^20^. In the meanwhile, the anti-correlation between the EASM and the EAWM is inconsistent with Chinese historical records during some key centennial periods in the last millennium^21^. Furthermore, this anti-correlation is now challenged by the reconstructed EAWM sea surface temperature (SST) indices in the South China Sea^15^,^16^ and lake-level changes in central Asia^18^, which suggest a positive correlation between the EAWM and the EASM in the last deglaciation on the orbital timescale. Another independent and robust evidence indicate that EAWM, reconstructed from loess grain sizes in Northwest China, was closely linked with Greenland's isotope records on millennial and glacial-to-interglacial timescales^18^, leaving the precessional-scale winter monsoon changes not well resolved because of the intrinsic feature of loess data. All the debate and inconsistence among proxy records raise a fundamental question: How is the EASM and EAWM correlated in response to past climate changes?

In this study, we revisit the co-variability of EASM and EAWM during the last 21,000 years by combining long-term transient model simulations and reconstructed indices. We conclude that a changing monsoon correlation, from a positive correlation at orbital timescale to a negative correlation at millennial timescale, was the key responses to solar insolation at northern hemisphere (NH) mid-latitudes and the fresh water fluxes in the North Atlantic. Greenhouse gases and continental ice sheets also have minor effects in modulating East Asia monsoons.

## Results

### Simulated and observed monsoons

**Figure 1:**
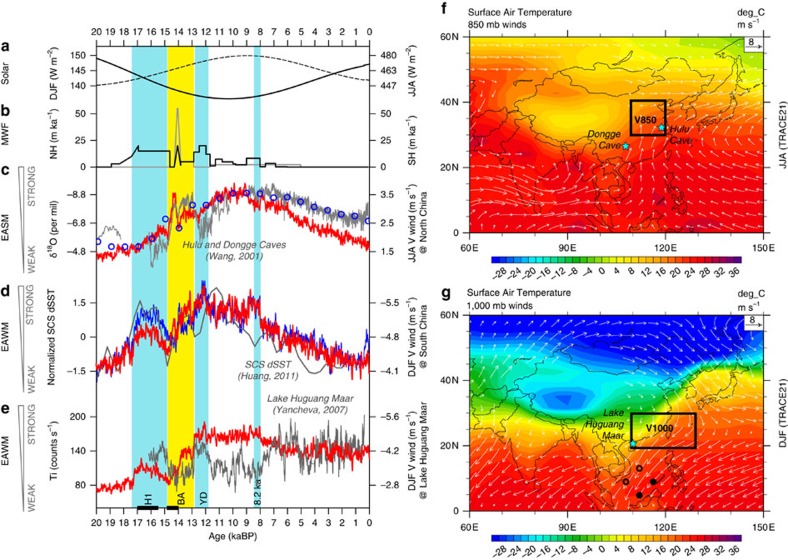
TRACE simulation and observations for EASM and EAWM in the last 20,000 years. (**a**) December-January-February (DJF) (solid line) and June-July-August (JJA) (dashed line) insolation at 45° N. (**b**) Melting water flux (in equivalent global sea level per 1,000 years) into the North Atlantic (black) and Southern Ocean (grey) in TRACE21. (**c**) δ^18^O (grey) from Dongge and Hulu caves (Wang *et al*.^3^), precipitation δ^18^O simulated in isotope-enable snapshot experiments (blue circles, Liu *et al*.^26^) and EASM wind index in TRACE21 (red). (**d**) SCS SST gradient index (dSST) for EAWM in the reconstruction (grey, Huang *et al*.^16^) and in TRACE21 (blue), EAWM wind index in TRACE21 (red). (**e**) Sediment Ti content from Lake Huguang Maar (grey) that was considered as an EAWM indicator by Yancheva *et al*.^14^, and the simulated meridional wind speed (red) near Lake Huguang Maar in TRACE21. (**f**) Modern June–August climatology of surface temperature and 850 mb winds in TRACE21, the locations for Hulu and Dongge caves (cyan stars), the model domain (110–120 E, 30–40 N) for calculating the EASM meridional wind index at 850 mb. (**g**) Modern December–February climatology of surface temperature and 1,000 mb winds in TRACE21, the location of Lake Huguang Maar (cyan star) and the sites for Huang's (2011) SST index (two solid and two open circles), and the model domain (110–130 E, 20–30 N) for calculating the EAWM meridional wind index at 1,000 mb. Three cold events, Heinrich 1 (H1), Younger Dryas (YD) and 8.2 ka, are marked as the blue vertical panels. The warm event Bølling-Allerød (BA) is marked as the yellow vertical panel.

Here we study the EASM–EAWM co-variability in a transient simulation of the climate evolution of the last 21,000 years (TRACE21) using the National Center for Atmospheric Research (NCAR) Community Climate System Model version 3 (CCSM3)^21^. TRACE21 is forced by the realistic climate forcing that consists of the orbital insolation, atmospheric concentration of greenhouse gases (GHGs), continental ice sheets and meltwater fluxes (Methods) and has been shown to simulate deglacial climate changes consistent with many proxy records at global and regional scales^21^,^22^,^23^,^24^. In particular, CCSM3 simulates both the EASM and EAWM reasonably well in the atmospheric wind field, with the EASM characterized by a southeasterly penetrating deep into northern China and the EAWM featured by a northeasterly sweeping across southern China and South China Sea (SCS; Fig. 1f,g and Supplementary Fig. 1).

TRACE21 simulates an EASM evolution largely consistent with the observation. Consistent with the classical view that a stronger EASM is accompanied by a deeper moisture penetration into northern China^25^, we will use the summer southerly averaged in East China as an index for the EASM in the model^26^ (Fig. 1f). Overall, the simulated EASM intensifies from the LGM (21 ka) towards the early Holocene (10 ka), and then weakens towards the late Holocene; this orbital scale response is punctuated by millennial events with an abrupt intensification at the Bølling onset and a subsequent weakening towards YD (Fig. 1c, red). This evolution pattern is largely consistent with the proxy records available^27^,^28^. The model-data consistency is reinforced by a direct comparison of the δ^18^O in East China between the cave records^3^,^4^,^5^ (Fig. 1c, black) and the isotope simulations^26^ (Fig. 1c, blue circles), the latter being derived from the atmospheric precipitation δ^18^O simulated in a series of 21 snapshot experiments in an isotope-enabled atmosphere model forced by the SST of TRACE21 (Methods).

TRACE21 also simulates an EAWM evolution in agreement with the observation in terms of a SST gradient index (Fig. 1d, red and black; Methods). Both the model and proxy EAWM SST indices show a distinct orbital scale response in phase with EASM, such that both EAWM and EASM intensify from LGM towards the early Holocene and then weaken towards the late Holocene. This positive EAWM–EASM correlation, however, seems to become negative during the millennial events of H1-BA-YD. This SST gradient index is derived with the southeastern SST subtracting the northwestern SST in the SCS. Physically, the EAWM northerly advects cold and dry continental air to the southwestern SCS along the Vietnam coast (Fig. 1g), forming a cold tongue and in turn a positive west-to-east SST gradient. A stronger SST gradient is therefore forced by a stronger EAWM northerly^15^,^16^. Our model also simulates a consistent SST gradient index and EAWM (blue and red in Fig. 1d), supporting the SST gradient as an index for EAWM. In contrast to the SST index that is determined by large-scale dynamics closely related with the EAWM, Lake Huguang Maar is located in a region of complex terrain and the lake deposition can be affected by the river's catchment and various local hydrological processes^20^. Our model also simulates diverse evolution patterns of the local wind surrounding the lake site, none of which is consistent with the Lake record throughout the last 21,000 years (Supplementary Fig. 2). The Lake Huguang Ti content shows a relatively consistent variability as compared with the SST index (Fig. 1d, blue) during the deglaciation, strengthening during H1, weakening in BA and strengthening again towards YD (Fig. 1e, black). However, after the YD towards the early-to-mid Holocene (12–8 ka), opposite to the maximum response in the proxy SST index and the model EAWM, the lake record declines to a minimum (Fig. 1e), destroying the positive correlation with EASM at orbital scale. We speculate that the linkage of lake record with the large-scale EAWM circulation might be interrupted by local hydrological processes after YD event.

### Responses to orbital and North Atlantic meltwater forcing

**Figure 2:**
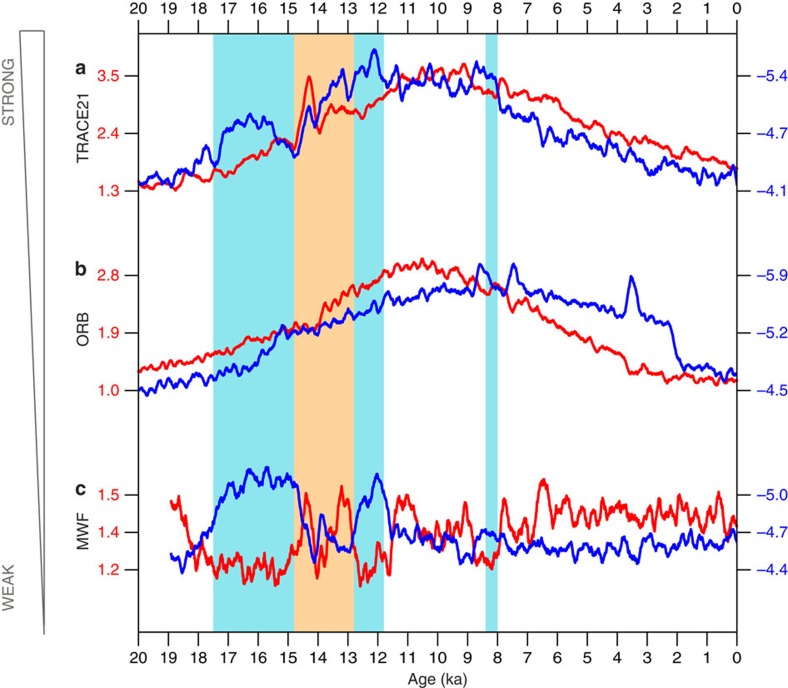
Monsoon index in TRACE and single forcing experiments. EASM (red) and EAWM (blue) wind indices in (**a**) TRACE21, and single forcing experiments (**b**) ORB and (**c**) MWF. The EAWM and EAWM indices are defined in Fig.1f,g after a 200-year running-average. Three cold events, Heinrich 1 (H1), Younger Dryas (YD) and 8.2 ka, are marked as the blue vertical panels. The warm event Bølling-Allerød (BA) is marked as the yellow vertical panel.

Our model-data comparison suggests a changing monsoon correlation, from a positive correlation at orbital timescale to a negative correlation at millennial timescale. The opposite monsoon correlations at orbital and millennial timescales, we hypothesize, is caused by different forcing mechanisms, with the former dominated by the precessional forcing, whereas the latter by the meltwater forcing. This hypothesis is confirmed in two sensitivity experiments that are integrated through the last 19,000 years the same as TRACE21, but forced by the orbital forcing (ORB) (Fig. 2b) and meltwater forcing (MWF) (Fig. 2c) individually (Methods). It is seen that the EASM and EAWM are largely correlated in experiment ORB (Fig. 2b), but anti-correlated in experiment MWF, especially for the major millennial events during H1-BA-YD (Fig. 2c).

**Figure 3:**
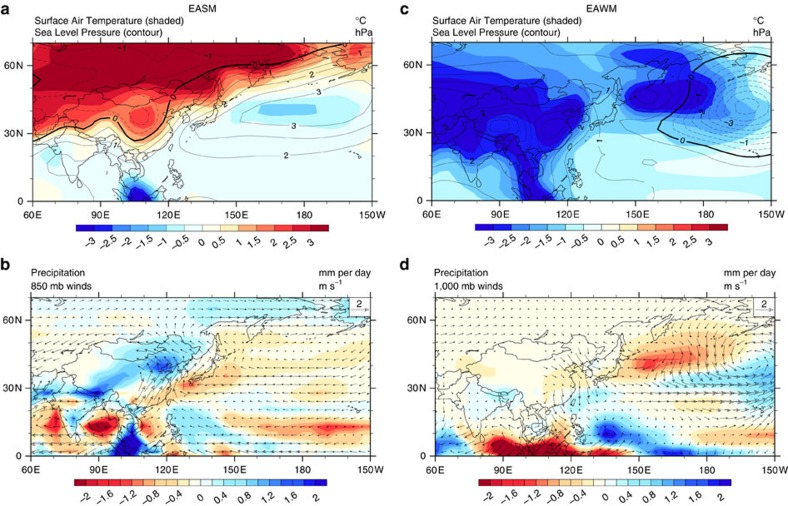
Monsoon response to insolation forcing on the orbital timescale. The differences of four key variables between the early Holocene (10–8 ka) and the late Holocene (2–0 ka) for EASM (**a**,**b**) and EAWM (**c**,**d**). Surface air temperature (colour) and sea level pressure (contour) are shown in **a**,**c**. Winds and total precipitation (colour) are shown in **b**,**d**.

The monsoon correlation at orbital scale is dominated by the classical monsoon response to precessional forcing^29^,^30^(Fig. 1a). Figure 3 shows the difference between the strong (10–8 ka) and weak (2–0 ka) EASM (EAWM) conditions in terms of the summer (winter) surface wind as well as the surface temperature, pressure and precipitation in TRACE21. The differences reflect mainly the orbital scale evolution (Figs 1 and 2) and can be discussed in the context of the slow evolution from the LGM towards the early Holocene^31^. As perihelion shifts from boreal winter to summer, the insolation is enhanced (reduced) in the NH in summer (winter). This leads to a warming (cooling) and lower (higher) pressure over the East Asia relative to the ocean and, in turn, a stronger southerly EASM (northerly EAWM) wind and increased precipitation downstream of the monsoon wind moisture transport (Fig. 3).

**Figure 4:**
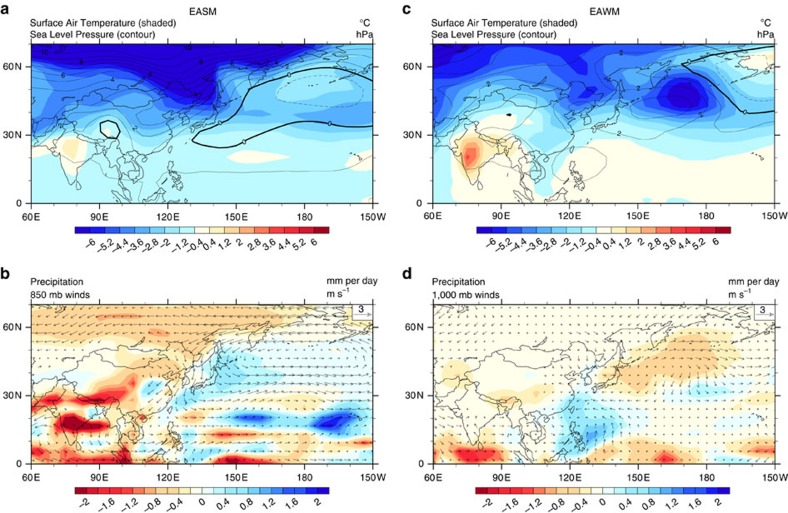
Monsoon response to melting water on the millennial timescale. Differences of key meteorological variables between H1 (17.0–15.5 ka) and Bølling-Allerød (14.8–14.0 ka) for EASM (**a**,**b**) and EAWM (**c**,**d**). Surface air temperature (colour) and sea level pressure (contour) are shown in **a**,**c**. Winds and total precipitation are shown in **b**,**d**.

In contrast, the anti-correlation monsoon response for millennial events is caused by the meltwater flux into the North Atlantic and the resulted change in the Atlantic Meridional Overturning Circulation (AMOC; Supplementary Fig. 3, ALL and MWF). A meltwater flux into the North Atlantic weakens the AMOC and the associated poleward oceanic heat transport, leading to a cooling that extends from the North Atlantic across the Eurasian continent throughout the year^21^,^32^. This response can be illustrated in the difference between H1 and BA. The colder northern China generates a higher pressure over land and in turn an anomalous northerly wind that weakens EASM (Fig. 4a,b), but enhances EAWM (Fig. 4c,d). The enhanced northerly in winter is somewhat weak in TRACE21, because of the effect of other forcing. Indeed, for experiments that are forced by the meltwater flux alone, the anomalous northerly wind becomes more significant in winter as well as summer (Fig. 2c and Supplementary Fig. 4).

### Responses to other minor forcing

The deglacial global climate evolution is also forced by rising GHGs (Supplementary Fig. 5a) and retreating ice sheets (Supplementary Fig. 5b). For the East Asia monsoon, however, the impact of GHG and ice sheet forcing seem to compensate each other. This is shown in two additional sensitivity experiments forced by the GHGs (CO2) and ice sheets (ICE) individually (Methods). The rising GHGs during the early deglacial period strengthen the EASM but weakens the EAWM, leading to an anti-correlation in experiment CO2 (Supplementary Fig. 5a,c). In contrast, the retreating ice sheets in experiment ICE lead to a weakening EASM and strengthening EAWM, also an anti-correlated monsoon response (Supplementary Fig. 5b,d), but with the opposite signs to that in CO2. As such, the effects of GHG and ice sheet tend to cancel out, leaving little residual after the deglaciation.

The anti-correlation monsoon response in experiment CO2 seem to differ from the monsoon response to future global warming, likely contributed by the indirect impact of the AMOC change. In response to future global warming, current climate models show a robust intensification of the EASM wind^1^,^33^,^34^,^35^,^36^ (Supplementary Fig. 6a), consistent with experiment CO2. This EASM intensification is caused by the direct radiative warming effect, which forces a stronger warming and lower pressure over land than over ocean and, in turn, an intensified southerly over East Asia (Supplementary Fig. 7). In comparison, there is no clear response of EAWM across these models^1^,^35^ (Supplementary Fig. 6b). The robust EAWM response and, in turn, the anti-correlation monsoon response in CO2 can be contributed by the strengthening AMOC^37^ in CCSM3 (Supplementary Fig. 3; Methods), which, opposite to the case of meltwater forcing (Fig. 2d and Supplementary Fig. 4), warms the Eurasian Continent, enhances the EASM, but weakens EAWM, contributing to the anti-correlation monsoon response. Finally, the anti-correlation monsoon response to ice sheet retreat seems to be caused by the shift of the westerly jet^38^ in response to the lowering ice sheets and their global climate impact (Methods).

## Discussion

Our study suggests that the evolution of EASM and EAWM is correlated at the orbital scale in response to the precessional forcing, but anti-correlated at millennial timescale in response to the meltwater forcing and the resulting AMOC change. The monsoon correlation is also affected by the rising GHGs and retreating ice sheets, but the two responses might be opposite such that there is little residual signal left after the deglaciation. Another, the current simulation might be too short to address the slow-varying impacts from ice sheets and GHGs. Their coupled effects over monsoon variability need more investigations on the glacial-interglacial timescale.

The new understanding here, from a modelling framework, has reconciled the conflicting interpretations on the co-variability between EASM and EAWM in proxy records, and demonstrated that their relationship is influenced by multiple physical processes on diverse timescales. Our findings indicate that any single correlation/anti-correlation, as concluded in previous studies, is not sufficient to describe the complicated nature of East Asia monsoon system. This work presents great challenge and potential to understand the response of the East Asian monsoon system to global climate changes in the past and the future.

## Methods

### TRACE21 and single forcing experiments

TRACE21 is a transient simulation of the global climate of the last 21,000 years in CCSM3 of the NCAR^39^ forced by the realistic climatic forcing, including orbital insolation^40^, atmospheric GHGs^41^, melting water discharge^42^ (Fig. 1b), the continental ice sheets derived from ICE-5G^43^ and the modification of coastlines and bathymetry at 13.1, 12.9, 7.6 and 6.2 ka for the Barents Sea, the Bering Strait, Hudson Bay and the Indonesian through-flow, respectively. More details can be found in ref. 44.

Four single forcing sensitivity experiments are also integrated the same as TRACE21, except that each sensitivity experiment is forced by only one single transient forcing with the other forcing fixed at their values at 19 ka. The ORB, CO2, MWF and ICE experiments are forced by the changing orbital insolation, GHG concentration, meltwater flux and the ice sheet, respectively^45^,^46^.

### Snapshot simulations in a water isotope enabled CAM3

For a direct model-data comparison of water isotopes, a set of time slice simulations are performed in NCAR Community Atmosphere Model (CAM) version 3 with the incorporation of a water isotope module (isoCAM3)^47^. We conducted 23 snapshot sensitivity experiments in the last 21,000 years: 21 experiments are 1,000 years apart, at 20, 19, …, 0 ka, and 2 experiments are at the time of special events, 14.5 ka (BA) and 12.1 ka (YD). Each slice is integrated for 50 years with the forcing of land-use, land-sea mask, sea surface temperature (SST) and sea-ice fraction that taken from TRACE21. The first 20-year results are removed as the spin-up and the remaining 30-year results are averaged for the analysis. A more complete description of these experiments can be found in Liu *et al*.^26^.

### Selection of EAWM index

The paleoclimatic proxies suggest that the EAWM index can be effectively derived from two major places. One is Northwest China^17^ and another is South China^14^,^16^. The winds in Northwest China mostly reflect the intensity of westerlies, which is more sensitive to AMOC rather than East Asian Meridional circulation. The winds in South China represents EAWM's intensity over North-to-South China and surrounding coastal region, as well as the land–sea sea level pressure gradient. Thus, we select the meridional wind at 1,000 hPa (V1000) over South China to serve as the EAWM index (Fig. 1g).

### Monsoon responses in experiments CO2 and ICE

Experiments CO2 and ICE show that the monsoon responses to the increasing GHGs and retreating ice sheets are the opposite and therefore tend to compensate each other. In CO2, EASM strengthens and EAWM weakens, generating an anti-correlation monsoon response. In ICE, EASM weakens and EAWM strengthens, also an anti-correlation monsoon response, but of the opposite sign to that in CO2 (Supplementary Fig. 5).

In response to increased GHGs in CO2, the strengthened EASM is consistent with the future global warming experiments in current climate models^1^ (Supplementary Fig. 6a). This robust EASM intensification is caused by the direct radiative warming effect, which forces a stronger warming and lower pressure over land than over ocean and, in turn, an intensified southerly over East Asia(Supplementary Fig. 7). In contrast, the weakening of EAWM wind in CO2 (Supplementary Fig. 6b, black dots) is no robust across various model in global warming experiments^1^,^36^ (Supplementary Fig. 6b). The difference in the EAWM responses between CO2 and global warming experiments, we speculate, is caused by an indirect impact of the AMOC change. In CO2, the AMOC is intensified in response to the slow rise of GHGs (Supplementary Fig. 3, CO2), which reduces sea ice cover and therefore increases the heat loss out of the glacial North Atlantic, and therefore intensifies the deep convection and in turn AMOC^37^. As discussed earlier on the monsoon response to meltwater forcing in Fig. 2d, the intensified AMOC warms the North Hemisphere continent, which weakens the EAWM, and reinforces the EASM, contributing to the anti-correlation monsoon response in CO2.

The causes for the responses of both EASM and EAWM to ice sheet retreat seem complex. In response to the lowering of ice sheets, especially the large reduction of Laurentide Ice sheet at ∼14 ka, the westerly jet shifts northward over the North Atlantic^48^, which leads to a cooling over the North Pacific because of the sea ice expansion^38^, an intensified Aleutian Low and a weakened western Pacific Subtropical High year round. (Supplementary Fig. 8a,c). This leads to a weakening of the EASM southerly wind and intensified EAWM northerly wind (Supplementary Fig. 8b,d), and in turn an anti-correlation monsoon response. In the meantime, AMOC is also weakened because of the sea ice expansion over the North Atlantic associated with the jet shift^39^ (Supplementary Fig. 3, ICE). The weakening AMOC should also contribute to a cooling over the Northern Hemisphere and in turn a weakening EASM and strengthening EAWM, contributing to the anti-correlation monsoon response in ICE.

### Data availability

The climate model data used in this study are available in Earth System Grid at NCAR, ‘ https://www.earthsystemgrid.org/project/trace.html'. The other data that support the findings of this study are available in Harvard Dataverse, ‘ http://dx.doi.org/10.7910/DVN/BMAG9U'.

## Additional information

**How to cite this article:** Wen, X. *et al*. Correlation and anti-correlation of the East Asian summer and winter monsoons during the last 21,000 years. *Nat. Commun.* 7:11999 doi: 10.1038/ncomms11999 (2016).

## Supplementary Material

Supplementary Information Supplementary Figures 1-8
