# Supplementary material for: Correlation and anti-correlation of the East Asian summer and winter monsoons during the last 21,000 years
Source: Nat Commun. 2016 Jun 22;7:11999. doi: 10.1038/ncomms11999 (PMC4917960; doi:10.1038/ncomms11999)
Supplement: Supplementary Information — Supplementary Figures 1-8 [file ncomms11999-s1.pdf]

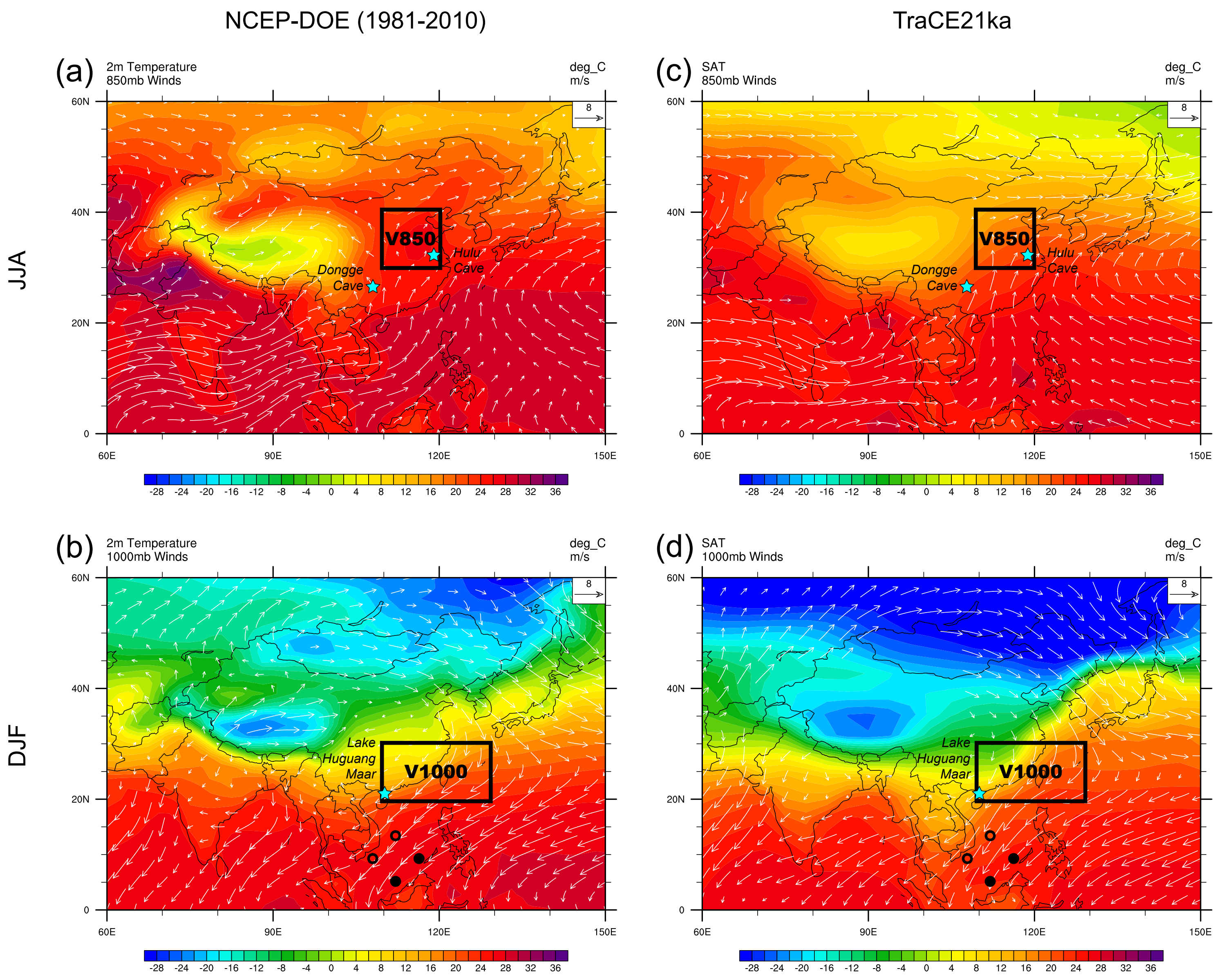

Supplementary Figure 1 | Climatology of EASM and EAWM. Observed and CCSM3 simulated climatology for summer (June-July-August, a and c) and winter (December-January-February, b and d) of surface air temperature and 1000mb (winter)/850mb (summer) winds. The stars indicate the locations of Hulu and Dongge caves, as well as Lake Huguang Maar. The solid and open circles indicate the sites for the SST-based EAWM index proposed by Huang (2011). The boxes indicate the domain for calculating the EASM and EAWM wind indices.

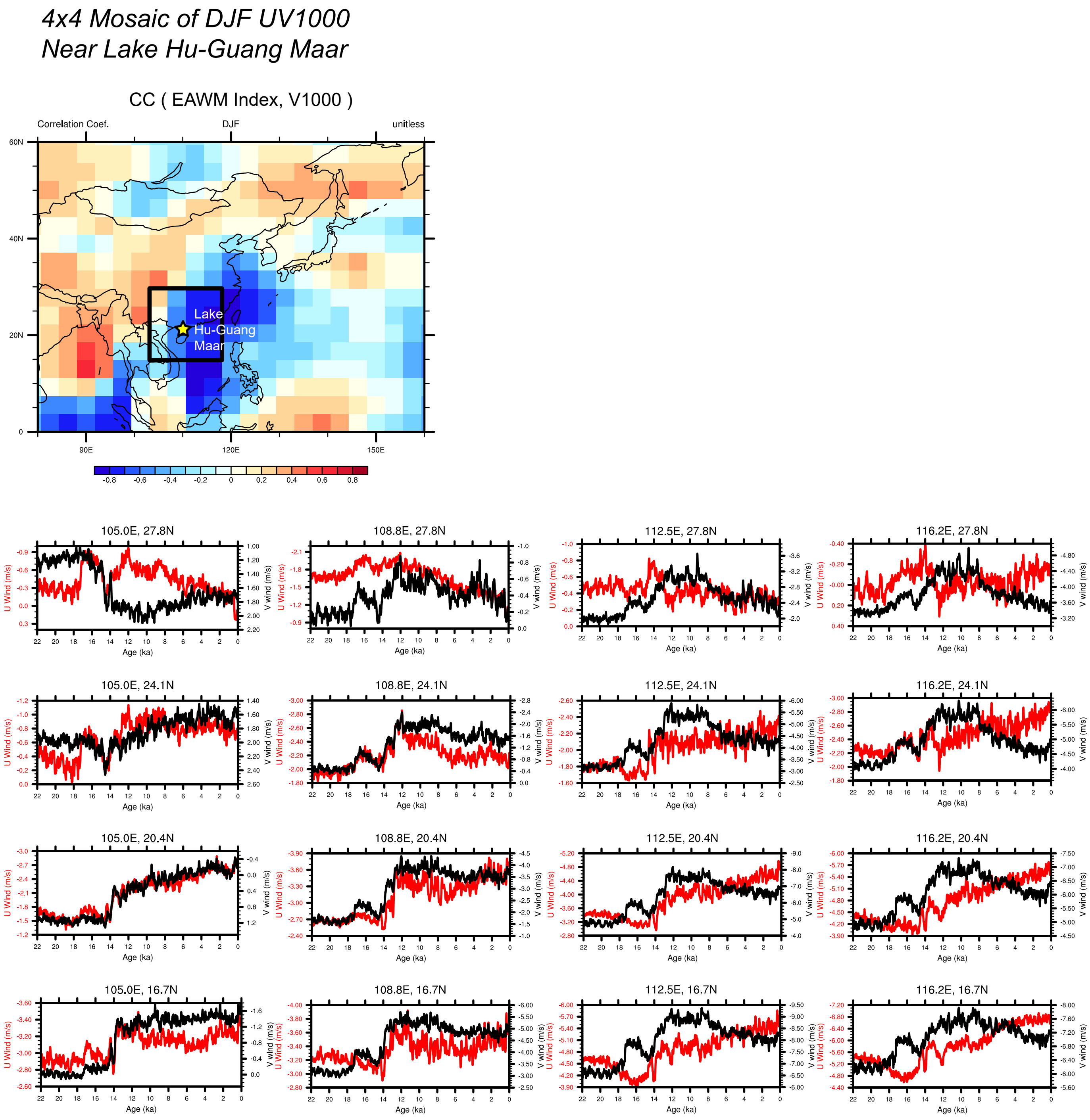

Supplementary Figure 2 | Mosaic wind evolution near Lake Huguang Maar. Time series of DJF zonal (U1000, red) and meridional (V1000, black) wind on the 4x4 model grid points surrounding Lake Huguang Maar, with the central longitude and latitude of each box labeled on the top of each subplot. The correlation between the modeled EAWM SST index and V1000 at each grid point is also shown on the left panel. It is shown that the wind evolution exhibits diverse patterns in different regions and between the zonal and meridional winds at current model grid scale (300-km x 300-km). In comparison with the Ti content in Lake Huguang Marr (Fig. 1e), the model winds near Lake Huguang Maar may reflect partly the EAWM strength, but none of the regional wind variation is consistent with the Ti content in the lake, as proposed by Yancheva (2007). We speculate that the Ti content in the sediments may be related to local surface winds at the lake site, instead of EAWM wind at regional and large scales.

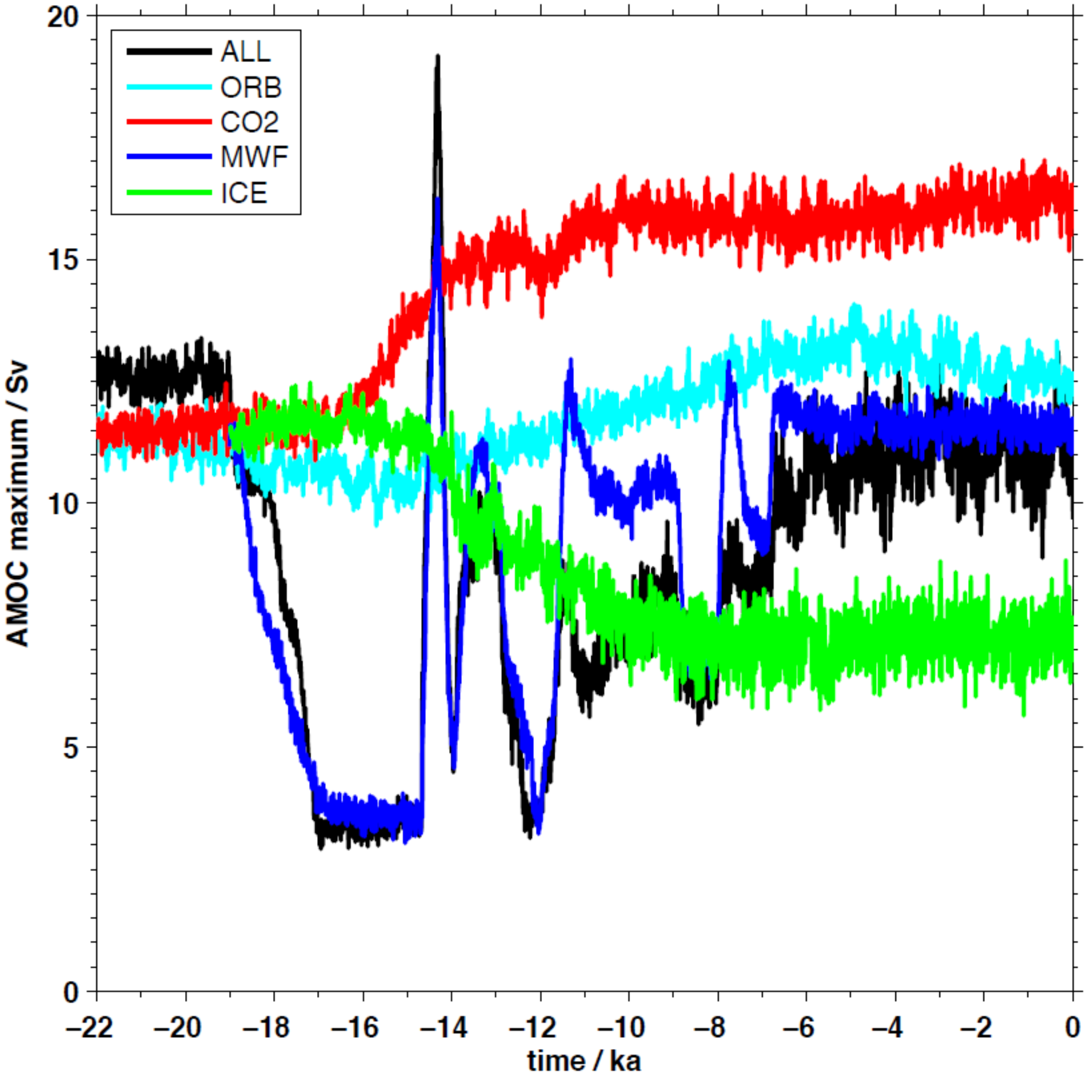

Supplementary Figure 3 | AMOC in TRACE and single forcing experiments. The evolution of AMOC transport (in unit of Sv), defined as the maximum in the Atlantic overturning streamfunction below 500-m, in the all forcing experiment TRACE21 (black) and four single forcing experiments (ORB in cyan, CO_2 in red, MWF in blue and ICE in green).

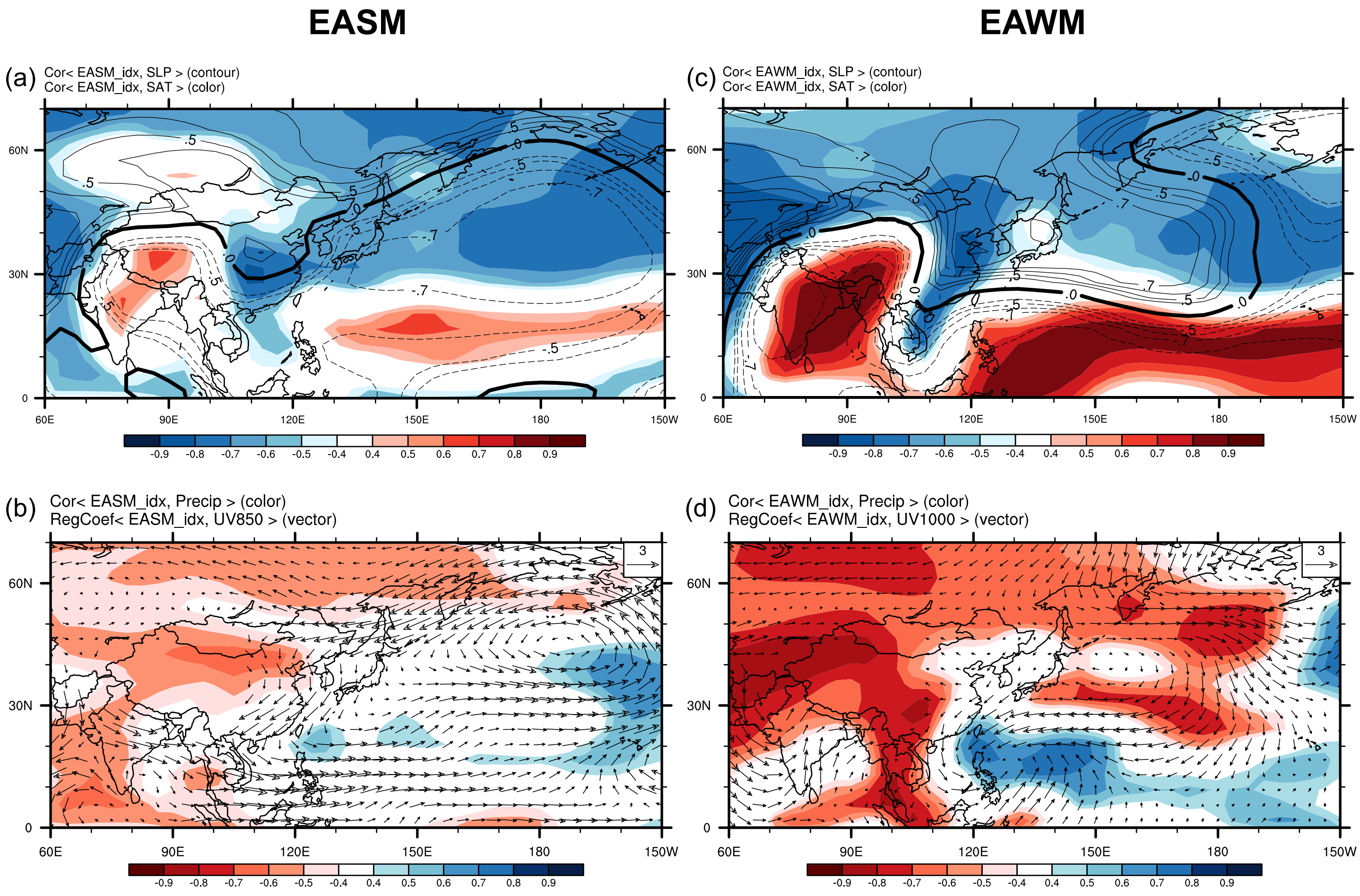

Supplementary Figure 4 | Monsoon response to melting water flux. The correlations between simulated wind index and several key meteorological variables are shown in (a) and (b) for EASM, (c) and (d) for EAWM, including sea level pressure (SLP), winds at 850 mb (UV850) and 1000 mb (UV1000), surface air temperature (SAT), and total precipitation.

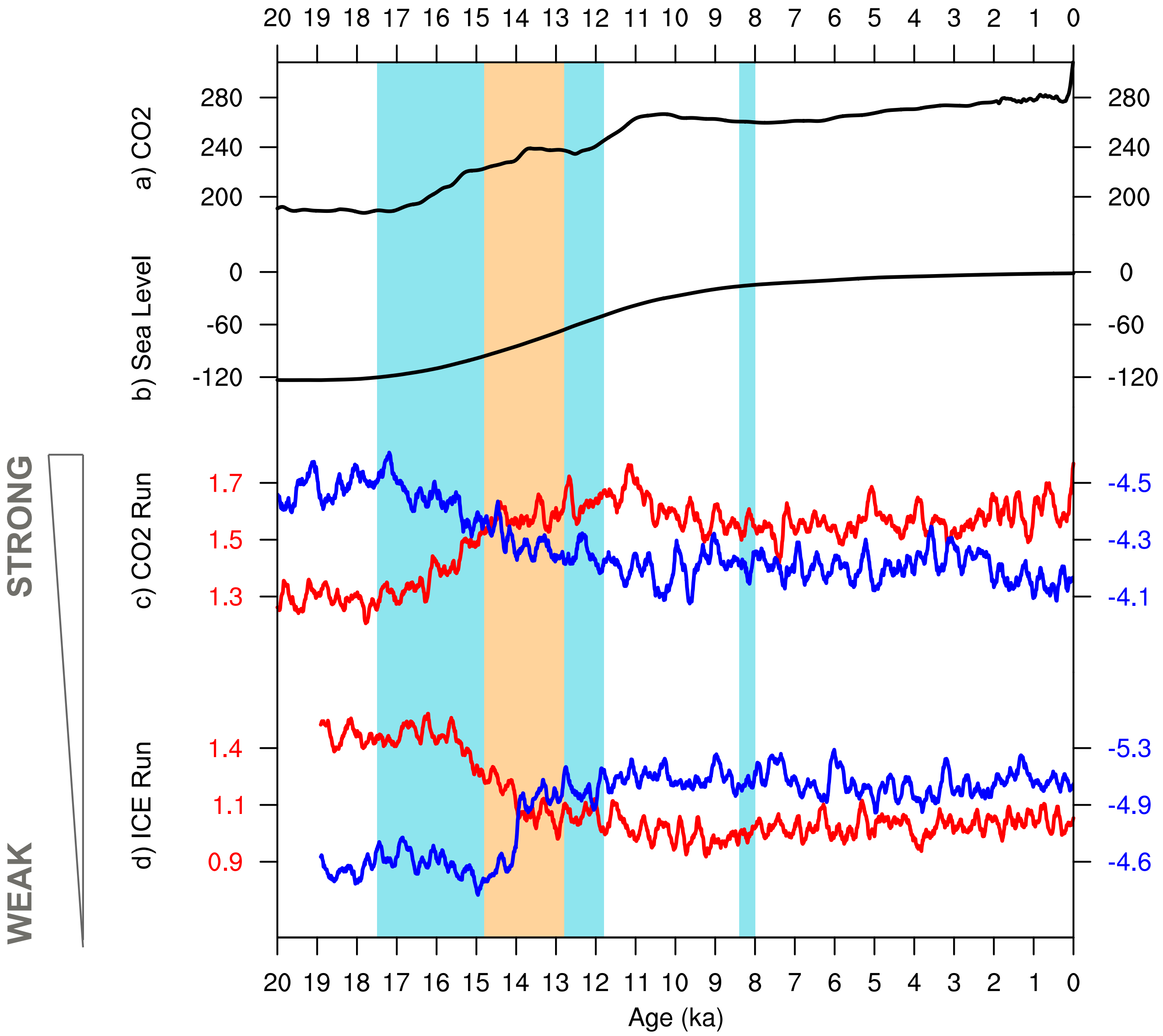

Supplementary Figure 5 | Monsoon index in experiments CO2 and ICE. The evolution of (a) atmospheric CO2 and (b) ice sheet volume (in equivalent sea level height) and EASM (red) / EAWM (blue) indices (as in Fig 2) in the single forcing experiments (c) CO2 and (d) ICE.

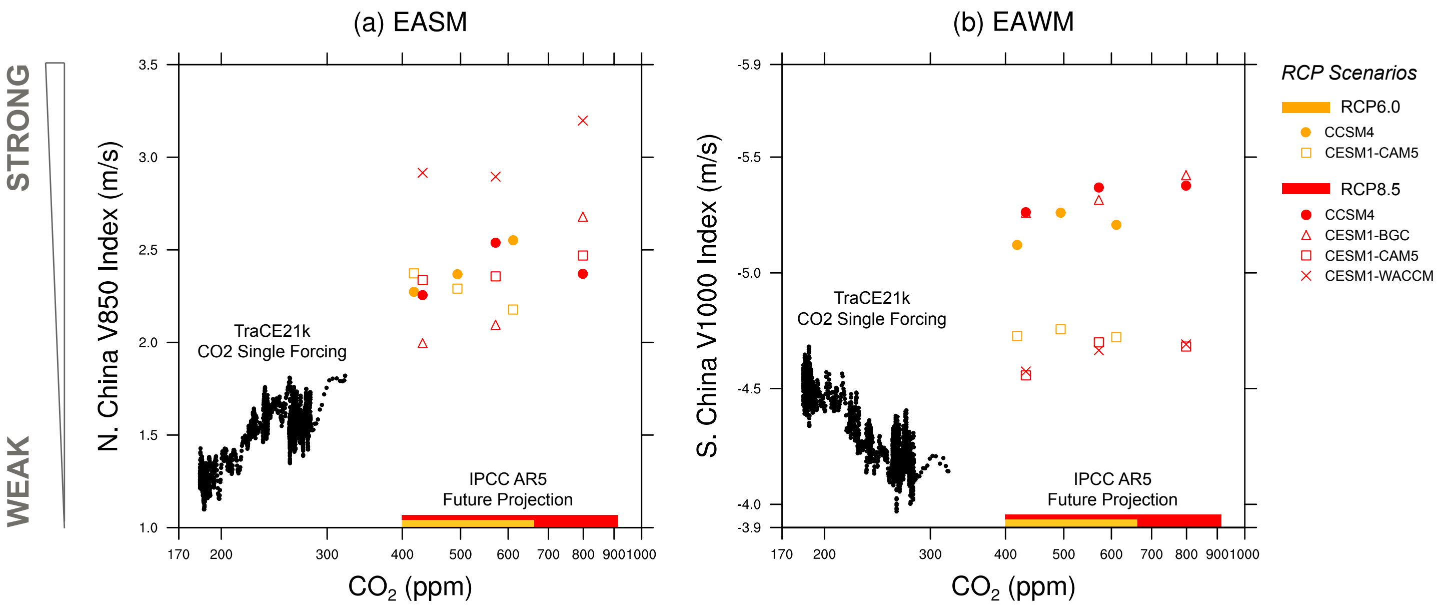

Supplementary Figure 6 | Monsoon response to CO_2 forcing in single forcing experiment and CMIP5 global warming experiments. The results show the response of the EASM (a) and EAWM (b) in the single forcing experiment CO2 and CMIP5 global warming experiments in CESM1 and CCSM4. The CMIP5 experiments are 2005-2100 simulations forced by increased atmospheric CO_2 concentration as prescribed in the future scenarios of RCP6.0 and RCP8.5. Each marker represents a 30-year mean in the CMIP5 experiments, and a 200-year mean in CO2 single forcing run. For the EASM, both experiment CO2 and CMIP5 experiments show a robust intensification with the rising CO_2. In comparison, the EAWM weakens in CO2 but shows no robust response in CMIP5 experiments.

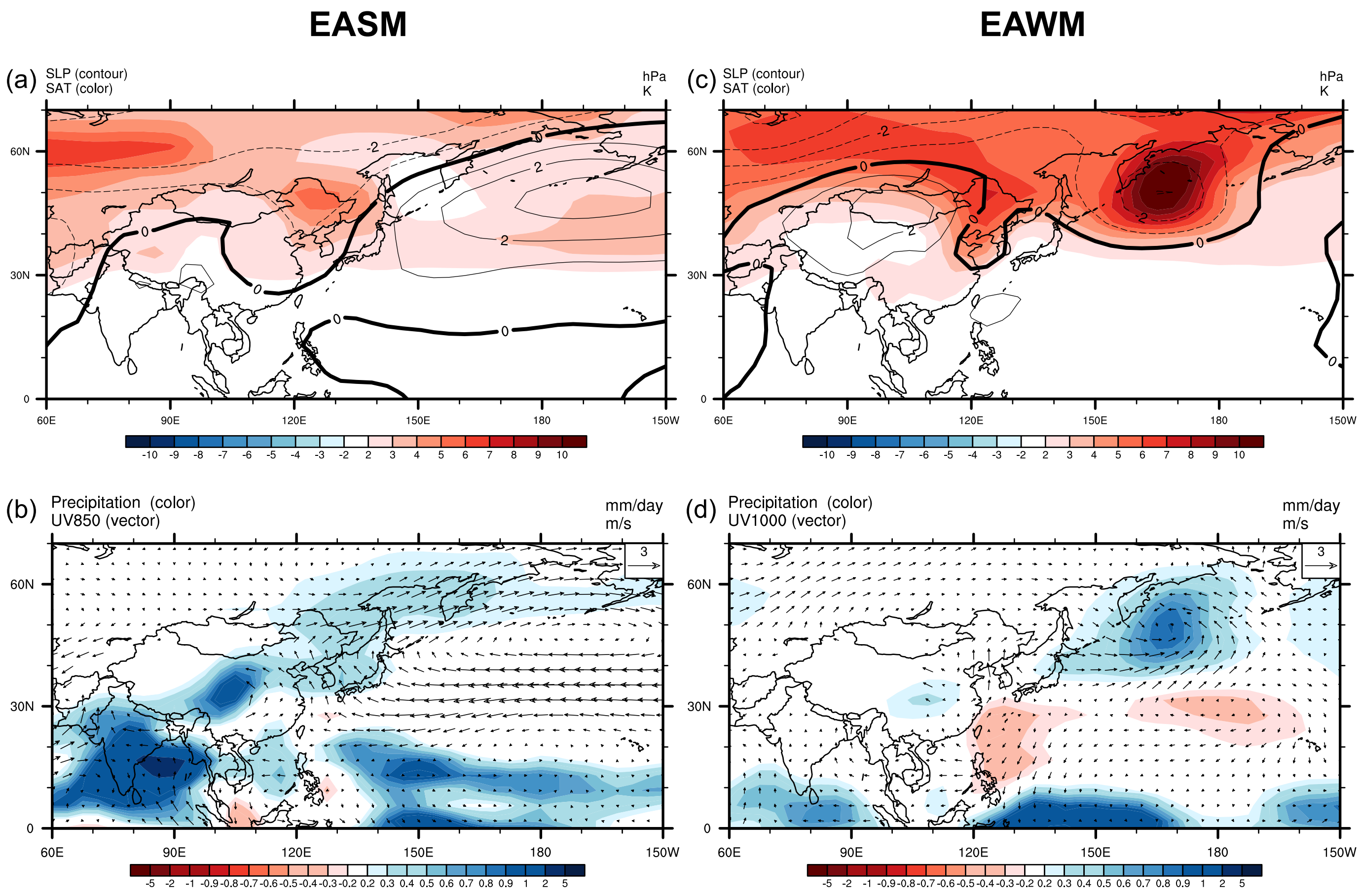

Supplementary Figure 7 | Monsoon response to CO2. Differences of key meteorological variables between 12-11ka and 18-17ka for EASM (a, b) and EAWM (c, d). Surface air temperature (color) and sea level pressure (contour) are shown in (a) and (c). Winds and total precipitation are shown in (b) and (d).

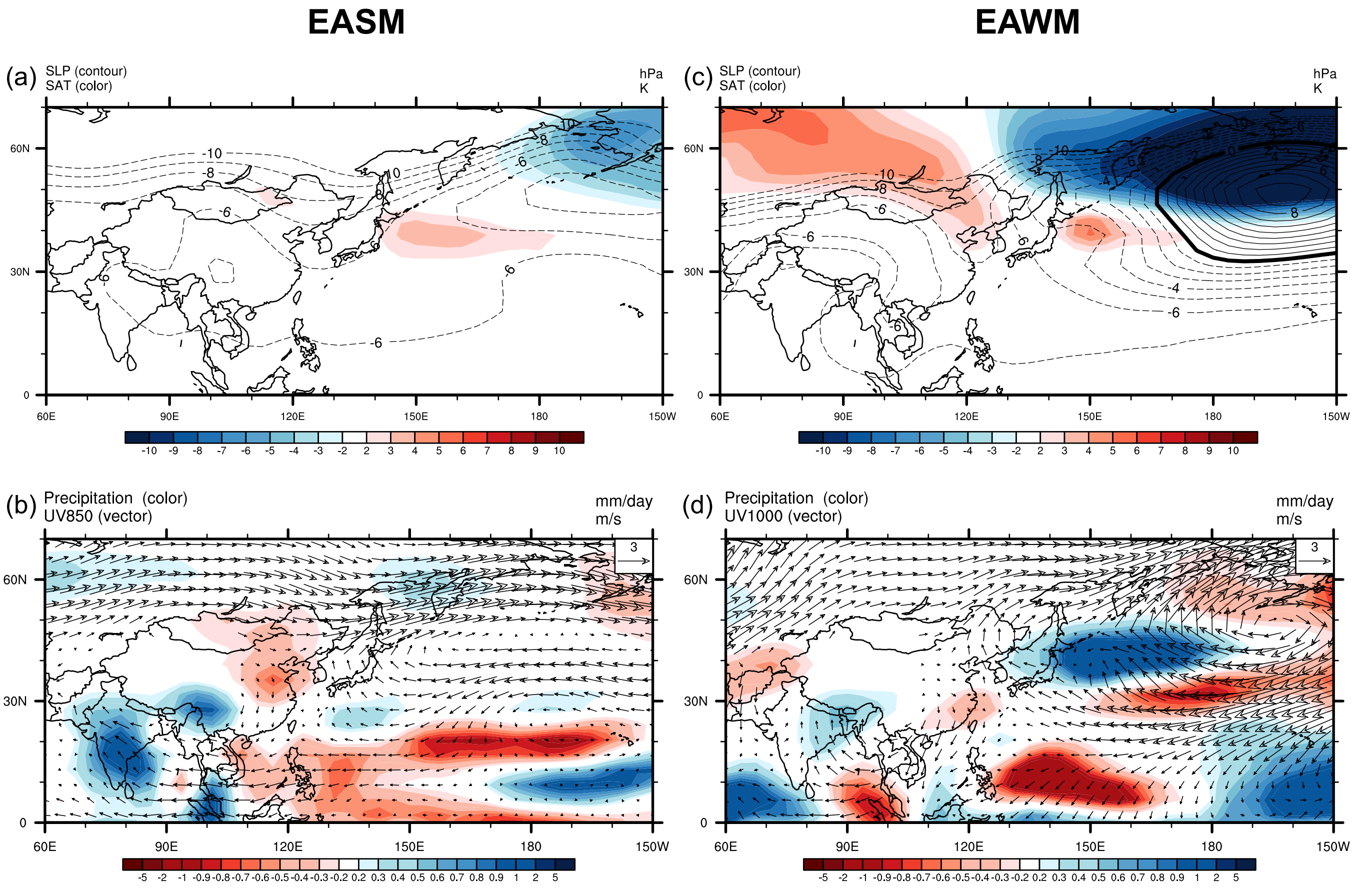

Supplementary Figure 8 | Monsoon response to ice sheet. Differences of key meteorological variables between 12-11ka and 18-17ka for EASM (a, b) and EAWM (c, d). Surface air temperature (color) and sea level pressure (contour) are shown in (a) and (c). Winds and total precipitation are shown in (b) and (d).
